# Book Reviews

**Published:** 1893-05

**Authors:** 


### *BOOK REVIEWS.*

---

A SYSTEM OF GENITO-URINARY DISEASES, SYPHILOLOGY AND DERMATOLOGY. By various authors. Edited by Prince A. Morrow, A. M., M. D., Clinical Professor of Genito-Urinary Diseases, formerly Lecturer on Dermatology in the University of the City of New York, etc., with illustrations, in three volumes. Vol. I., Genito-Urinary Diseases. New York. D. Appleton & Co. 1893.

As this work (Vol. I.) alone contains over a thousand pages, a review must necessarily be limited to the general plan of the book.

The leading authorities in the United States upon these subjects have contributed to the volume, each upon some special topic. We find the following subjects treated in the fullest manner: Anatomy and Physiology of the Genito-Urinary Organs; Diseases of the Penis; Diseases and Injuries of the Urethra; Etiology of Urethritis; Acute Urethritis—Gonorrhœa; Chronic Gonorrhœa or Gleet; Endoscopy; Gonorrhœal Ophthalmia; Gonorrhœal Rheumatism; Gonorrhœa of the Rectum, Nose, Mouth, Ear, Umbilicus and Axilla (a unique chapter); Stricture of the Urethra; Diseases of the Prostate; The Functional Disorders of Micturition; The Diagnostic Significance of Pathological Modifications in the Urine, including the most practical methods of Urine Analysis; Urinary Fever; Cystoscopy; The Cystites; Injuries and Diseases of the Bladder; Rupture of the Bladder; Stone in the Bladder, Prostate, Urethra and Ureters; The Surgical Diseases of the Kidney; Tuberculosis Uro-Genitalis; Diseases of the Scrotum; Diseases of the Testicle; Hydrocele and Spermatocele; Varicocele; Diseases of the Seminal Vesicles; Functional Disorders of the Male Sexual Organs; Gonorrhœa in the Female.

This array of subjects shows the cyclopædic character of the work. Dr. Morrow, the editor, has had charge of the arrangement of the matter and has also contributed to it. His training and experience eminently fit him for the work. The publishers have done their part well, as is characteristic.

It would seem that the owner of this volume would find in its pages all he would desire upon the subject. M. B. H.

---

HISTORY OF THE LIFE OF DR. D. HAYES AGNEW, M. D., LL. D. By J. Howe Adams, M. D. In one large Royal Octavo volume, 376 pages, Extra Cloth, beveled edges, $2.50 net. Half-Morocco, gilt top, $3.50 net. Sold only by subscription. Philadelphia. The F. A. Davis Co., publishers, 1914 and 1916 Cherry street.

The friends of the late Dr. Agnew all over the country will be glad to know that Dr. Adams has prepared this excellent history of his life. America probably never had a greater surgeon, or one who was more highly and universally esteemed. The story of his life is admirably told in this book. His professional career did not begin under flattering auspices, so he went into a business venture and failed. It was a fortunate failure. His long connection with the Philadelphia School of Anatomy, which was unquestionably one of the greatest factors in his subsequent success, is described in some detail; also his connections with the University of Pennsylvania as Professor of Surgery, and with several hospitals as attending and consulting surgeon. The author also details many incidents from his private life which show the man to have possessed a personality and charm of manner seldom equaled. He was a great worker, and in spite of the great demands upon his time made many contributions to medical and surgical literature. The book contains fourteen full page portraits taken at different periods of his life. This biography is the record of a life nobly and successfully spent and could be read with profit and inspiration by any physician.

---

ACNE AND ALOPECIA. By L. Duncan Bulkley, M. D.

This little book is published by Geo. S. Davis, of Detroit, in his Physician's Leisure Library Series. Dr. Bulkley is one of the leading dermatologists in New York, or in the United States for that matter. He explains publishing the two conditions in one book by their great frequency and the difficulty the general practitioner experiences in their management.

Acne, its varieties, causes and treatment, as well as the points of diagnosis, is first taken up and treated in that crisp, clear manner which is characteristic of the writer. He attaches much importance to internal treatment and the dietary in these cases, which, in fact, he does in most skin diseases.

Under Alopecia he describes the various kinds of loss of hair, both symptomatic and idiopathic, preceding this description with a brief *résumé* of the physiology and pathology of the hair. The little work is convenient, costs only 25 cents as published, and would be a valuable addition to the library of any physician. M. B. H.

---

THE DISEASES OF INEBRIETY; Its Pathology, Etiology, Treatment and Medico-Legal Relations. $2.75. E. B. Treat, 5 Cooper Union, New York.

This work is a collection of papers that have been presented from time to time to the American Association for the Study and Cure of Inebriety. This Association was organized about twenty-three years ago, and has been very industrious in its study of this question. The Association believes that inebriety is a disease curable as other diseases. This fact, the Introduction says, has passed beyond question, and hence all moral theories or discussions are of little or no value from a scientific point of view. Most of these papers have been published before in the Association organ, the *Quarterly Journal of Inebriety.* We think the authors' names should be attached. The compilation has been done by the energetic secretary, Dr. T. D. Crothers, of Hartford, Connecticut. It is a full and complete treatment of this great subject in all its phases. The inebrieties of opium, cocaine, ether, etc., are also discussed.

---

SYPHILIS AND THE NERVOUS SYSTEM, being a revised reprint of the Lettsomian lectures for 1890, delivered before the Medical Society of London, by W. R. Gowers, M. D., F. R. C. P., F. R. S., Consulting Physician to University College Hospital, Physician to the National Hospital for the Paralytic and Epileptic, etc. Philadelphia. P. Blakiston, Son & Co., 1012 Walnut Street.

To do this small volume justice, more words would be required than we can give to it here. Dr. Gowers has, in his masterly manner, opened wide this most interesting subject, and in equally as masterly a manner, dealt with it; and though he used but comparatively few words, yet this little volume is equal in information to any we have ever read.

These three lectures are models for brevity, lucidity, comprehensiveness, original thought, thorough investigation. H. H.

---

INTERNATIONAL MEDICAL ANNUAL AND PRACTITIONER'S INDEX. A Book of Reference for Medical Practitioners. 1893. Eleventh year. $2.75. E. B. Treat, 5 Cooper Union, New York.

Of many works of this character that are issued every year this is one of the best. It is well edited by an able corps of American and foreign writers, many of whom have made excellent contributions themselves. All departments of medicine and surgery are reviewed as thoroughly as circumstances would permit, and full mention has been made to all the more important contributions to our medical and surgical progress. It is an excellent digest of the contributions to the medical press, and, we think, a very serviceable work of reference for the busy physician.

---

THE YEAR-BOOK OF TREATMENT FOR 1893. A Critical Review for Practitioners of Medicine and Surgery. Philadelphia, Lea Brothers & Co.

*The Year-Books* of Lea Brothers are well known to the profession. The present one carries out the design of its predecessors, supplying an excellent review of medical and surgical progress during the past year. In this issue separate space is devoted to anæsthetics, and also to public health and hygiene. This is the ninth issue of the series.

---

LIPPINCOTT'S MAGAZINE FOR MAY, 1893.

The many admirers of Rosa Nouchette Carey will be gratified to learn that the complete novel in the May number of *Lippincott's* is from her facile and well-tried pen. Its title is "Mrs. Romney." The third in the series of Lippincott's Notable Stories, "A Pastel," by Cornelia Kane Rathbone, is a delicate and touching sketch of wasted loyalty and disappointed hope. It is illustrated throughout. James Cox furnishes a full and glowing account of "New St. Louis," illustrated with cuts of a dozen of the huge buildings which have lately risen in that thriving and progressive city. John Bunting traces the origin and history of "The Society of the Cincinnati," with the violent objections which were raised in its early days against its supposed aristocratic character and dangerous tendency. This article also is illustrated. Mrs. Gertrude Atherton supplies a short but appreciative account of the American sculptress, Kuhne Beveridge, with a cut of her most notable work, "The Sprinter." Professor L. M. Haupt has a brief article on "Colonel Pope and Good Roads." M. Crofton, in "Men of the Day," gives sketches of William Morris, the poet, Archbishop Satolli, and Secretary of War Lamont." The poetry of the number is by Louise Chandler Moulton, Dora Read Goodale, Charlotte Pendleton, and Arthur D. F. Randolph.

---

No. 2543 of *Littell's Living Age* completed the 196th Quarterly Volume, and the 49th year of the continuous publication of this excellent magazine. The volume covers the months of January, February and March, and its table of contents shows it to contain 97 articles, beside miscellany and poetry. These articles cover a wide range of topics, including valuable biographical and historical papers, readable essays and reviews, choice fiction, the latest results of scientific inquiry, sketches of travel and adventure, etc., etc. Each issue brings its weekly addition to this feast of good things. The early issues of the new volume which usher in its year of jubilee are not a whit lacking in any respect from their predecessors. The Inadequacy of Natural Selection, by Herbert Spencer; Aspects of Tennyson, Part III., by Agnes Lambert; A King's Treasurer, by H. C. MacDowell; A Packet of Old Letters, by Mrs. Andrew Crosse; Venetian Melancholy, by J. Addington Symonds; The Military Courage of Royalty, by Arch. Forbes, the great war correspondent (this is a reply to E. B. Lanin's paper on the Czar Alexander III., in the January number of *The Contemporary,* reprinted in No. 2537 of *The Living Age*); Scandal about Queen Elizabeth, by A. Lang;

Trained Workers for the Poor, by Miss Octavia Hill, whose name is as widely known and as highly revered by the workers among the poor of America as by those of England. These are only a few of the many articles of equal merit which go to make up the first three issues in April. It is impossible to find elsewhere so much that is valuable at so small a cost. For only $8.00 a year the readers of *The Living Age* have the cream of the whole British periodical press served them and that with a freshness and fullness, owing to its frequency of issue, not otherwise obtainable. Send 15 cents for a specimen copy to the publishers, Littell & Co., 31 Bedford St., Boston.

---

While writing with all the scientific knowledge of a great astronomer, Camille Flammarion in his marvellous story "Omega; The End of the World," which begins in the April number of *The Cosmopolitan* magazine, keeps the reader at the highest point of excitement by his vivid description of the alarm and despair excited by the approach of a comet whose collision with the earth had been declared by astronomers inevitable. The description begins at a time when the business of the world has been suspended, and at a great mass-meeting held in the Institute of France, we hear the discussion of scientists as to the possibility of a second deluge, the drying up of all the surface water of the globe, or the total destruction of human life by cold, together with all the possible phases of death paralleled by the history of the moon. For scientific statement and sensational effect this characteristic production of French genius is unique, and the reader who reads this marvellous story—and if he begins it he will certainly finish it—will have assimilated without effort, a compact store of scientific knowledge. In this way, apart from its absorbing interest, this remarkable piece of fiction will have a distinct scientific value.

